# YAP1 inhibits RSL3-induced castration-resistant prostate cancer cell ferroptosis by driving glutamine uptake and metabolism to GSH

**DOI:** 10.1007/s11010-023-04847-4

**Published:** 2023-09-29

**Authors:** Xian Fu, Hongshen Wu, Changjiu Li, Gang Deng, Chao Chen

**Affiliations:** Department of Urology, Affiliated Hangzhou First People’s Hospital, Zhejiang University School of Medicine, Hangzhou, China

**Keywords:** YAP1, Castrate-resistant prostate cancer, GPX4, Ferroptosis

## Abstract

High levels of YAP1 and ferroptosis activation in castration-resistant prostate cancer (CRPC) can inhibit CRPC progression and improve its sensitivity toward chemotherapeutics drugs. However, whether YAP1 regulates ferroptosis in CRPC cells and the underlying mechanisms are unknown. The protein levels of YAP1, SLC1A5, and GLS1 in benign prostatic hyperplasia (BPH), prostate cancer (PCa) that did not progress to CRPC, and CRPC tissue samples were evaluated using western blotting. In PC-3 and DU-145 cells, YAP1 overexpression vector, small-interfering RNA, specific inhibitor verteporfin, ferroptosis-inducer RSL3, SLC1A5-inhibitor V-9302, and GLS1-inhibitor CB-839 were used. Immunofluorescence, flow cytometry, dual-luciferase reporter gene, and related kits were used to investigate the effect of YAP1 on the ferroptosis activity in CRPC cells and its underlying mechanisms. YAP1 promoted extracellular glutamine uptake and subsequent production of glutamate and glutathione (GSH), and increases the GPX4 activity. For the activation of ferroptosis by RSL3, YAP1 decreased the levels of reactive oxygen species, malondialdehyde, and lipid peroxidation, and the proportion of dead cells. Mechanistically, YAP1 promoted the expression of SCL1A5 and GLS1 and further increased the GSH levels and GPX4 activity. Thus, inhibiting SLC1A5 or GLS1 activity could alleviate the antagonistic effect of YAP1 on the ferroptosis of RSL3-induced CRPC cells. In CRPC, the YAP1 level is high, which enters the nucleus and promotes the expressions of SLC1A5 and GLS1, thereby promoting cellular glutamine uptake and metabolism to generate glutamate and further synthesizing GSH, increasing GPX4 activity, improving cellular antioxidant capacity, and inhibiting cell death.

## Introduction

Prostate cancer (PCa) is a malignant tumor that occurs in the prostate epithelium. It is the most common tumor in the male urinary system. The global cancer burden data released by the International Agency for Research on Cancer (IARC) in 2020 reported 1.41 million new cases of PCa worldwide, including 120,000 cases in China. The report revealed that the incidence of PCa in China is increasing [1]. Low-grade PCa can be treated with radical prostatectomy or radiotherapy and high-grade or metastatic PCa can be treated with androgen-deprivation therapy (ADT). However, most of these patients progress to castration-resistant prostate cancer (CRPC) within 1–3 years, which is the main cause of death in patients with PCa. Although new androgen receptor (AR) inhibitors, chemotherapeutic drugs, and immunomodulatory have been developed in the past decade, their efficacy is limited. Therefore, new therapeutic drugs and strategies are required. Herein, we aimed to elucidate the pathological mechanism underlying CRPC.

Ferroptosis is an iron-dependent cell death caused by the accumulation of lipid peroxidation, which is different from apoptosis, necrosis, and pyroptosis. Fe^2+^- and reactive oxygen species (ROS)-driven increased lipid peroxidation, system Xc^−^-mediated cysteine import, glutathione (GSH) synthesis, and GPX4 activity deficiency are its main underlying molecular mechanisms [2, 3]. Ferroptosis is closely related to many biological functions. Notably, the knockout of 2,4-dienoyl-CoA reductase induces endoplasmic reticulum (ER) stress and sensitizes CRPC cells to ferroptosis [4]. Flubendazole, an FDA-approved anthelmintic, induces P53 expression, inhibits SLC7A11 transcription, and then downregulates GPX4, thus promoting ferroptosis in CRPC [5]. Ferroptosis inducers, such as erastin and RSL3, can inhibit the expression and activity of full-length AR and spliceosome AR in CRPC. The combination of erastin or RSL3 with the second-generation antiandrogen, enzalutamide, or abiraterone, gives a synergistic effect [6]. Thus, regulating ferroptosis can help in the treatment of CRPC and further reveal the regulatory mechanism underlying ferroptosis in CRPC, which will provide ideas and directions for the development of more effective therapeutic strategies.

Glutamine is the most abundant amino acid in human blood. It promotes the growth and metastasis of various tumors, including PCa [7, 8]. CRPC exhibits a stronger capacity for glutamine uptake and metabolism [9]. The AR is continuously activated in CRPCs, which can regulate the expression of glutamine uptake-regulated molecules and metabolism, and proto-oncogene-expressed proteins in CRPCs can induce the same metabolic changes [10, 11]. Notably, previous studies have found that there is a relationship between glutamine metabolism and ferroptosis activity. The main mechanism is that glutamine generates glutamate under the action of glutaminase 1(GLS1) and glutamate, and cysteine can generate *γ*-glutamylcysteine under the action of glutamate–cysteine ligase (GCLM). Glycine is added to generate GSH under the action of glutathione synthetase (GSS). GSH is a cofactor for GPX4, which is an important antioxidant molecule in ferroptosis. However, the mechanism underlying how glutamine uptake and metabolism to GSH occur in CRPC and their effects on ferroptosis are unclear.

YAP1 is a key molecule downstream of the Hippo pathway, which is regulated by mammalian Ste20-like kinases 1/2(MST1/2) and large tumor suppressor 1/2(LATS1/2). When the Hippo pathway is turned on, MST1/2 forms a complex with the Salvador family WW domain-containing protein 1(SAV1) to phosphorylate and activate LAST1/2. LAST1/2 binds to adaptor protein monopolar spindle-one-binder 1 and phosphorylates YAP, inactivating YAP, and degrading it by the proteasome in the cytoplasm. When the Hippo pathway is turned off, unphosphorylated YAP translocates to the nucleus to interact with transcription factors, such as TEA-domain transcription factor 1–4 (TEAD 1–4), to induce the expression of target genes [12]. We found that YAP1 promotes the progression of PCa. In CRPC, the upregulated expression of YAP1 promotes growth and metastasis, regulates AR target gene expression, and also promotes the transformation of androgen-sensitive PCa to insensitive PCa, which can improve cell stemness [13–17]. Recent studies have shown that YAP1 does not always induce ferroptosis, it can also inhibit ferroptosis and its roles in ferroptosis are cell specific [18]. However, the effect of YAP on the ferroptosis sensitivity of CRPC is unknown. YAP1 can promote the expression of SLC1A5 and GLS1 and promote glutamine uptake and metabolism to glutamate [19, 20]. However, no study has reported its role in CRPC. It is unknown whether YAP1 increases the GSH level and GPX4 activity of CRPC cells by regulating glutamine uptake and metabolism to glutamate and regulates the ferroptosis sensitivity of CRPC cells. In this study, clinical samples were analyzed to evaluate the levels of YAP1. In vitro CRPC cell lines were used to determine the effect of YAP1 on glutamine uptake and metabolism to GSH and RSL3-induced ferroptosis. We further elucidated the mechanism of YAP1 in regulating the ferroptosis sensitivity of CRPC. We found that YAP1 drives glutamine uptake and metabolism to GSH by regulating SLC1A5 and GLS1 expression and then inhibits lipid peroxidation and regulates cell death.

## Materials and methods

### Cell culture

PC-3 cells were purchased from Wuhan Procell Life Science & Technology Co., Ltd. and cultured in Ham's F-12K (Procell, China, PM150910) medium supplemented with 10% FBS (Procell, 164210-500). DU145 cells were purchased from Wuhan Procell Life Science & Technology Co., Ltd. and cultured in MEM (Procell, PM150410) medium supplemented with 10% FBS (Procell, 164210-500). The cells were cultured under the conditions of 37 °C, 5% CO_2_, and passaged at a ratio of 1:3.

### Cell treatment

The cells were treated with RSL3 (MCE, China, HY-100218A) at a concentration of 5 μM for 24 h. Verteporfin (MCE, HY-B0146) was used to treat the cells at a concentration of 1 μM for 24 h. V-9302 (MCE, HY-112683) was treated at a concentration of 20 μM for 48 h. CB-839 (MCE, HY-12248) was treated at a concentration of 1 μM for 48 h.

### Tissue sample

Benign prostatic hyperplasia (BPH), PCa, and CRPC tissue samples were collected from patients undergoing surgery at the Hangzhou First People's Hospital. All operations in this study were approved by the Medical Technology Clinical Application and Research Ethics Committee of Hangzhou First People's Hospital (approval number: KY-20211105-0066-01), and each participant's written informed consent was obtained.

### Transfection

The experiment was performed as per the standard protocol. Briefly, cells in the logarithmic growth phase were inoculated and cultured overnight in an incubator at 37 °C and 5% CO_2_. The medium was changed to serum-free medium 2 h before transfection. Then, 2 μL of YAP1 OE or vector plasmid (or 20 μM small-interfering RNA [siRNA] solution, Sangon, China) was diluted with 100 μL of serum-free opti-MEM, mixed gently, and kept for 5 min. Further, 3 μL of Lipofectamine™ 2000 (Invitrogen, China, 11668030) was diluted using 100 μL of opti-MEM and kept for 5 min. Lipofectamine™ 2000 and plasmid or siRNA were mixed and kept for 15 min. The mixture was added to each culture well, and after mixing, cells were cultured in an incubator at 37 °C and 5% CO_2_. After 6 h, the mixture was aspirated and replaced with a normal medium to continue culturing for 72 h, after which the cells were collected. The CDS region of NM_001130145.2 was selected for the YAP1 OE sequence. The YAP1 siRNA and NC siRNA sequences are shown in Table 1.

**Table 1:** YAP1 siRNA and NC siRNA sequences

	Sense (5′–3′)	Antisense (5′–3′)
YAP1 siRNA1	GCAUCUUCGACAGUCUUCUTT	AGAAGACUGUCGAAGAUGCTT
YAP1 siRNA2	GGUCAGAGAUACUUCUUAATT	UUAAGAAGUAUCUCUGACCTT
YAP1 siRNA3	GGUAGCGCUUUGUAUGCAUTT	AUGCAUACAAAGCGCUACCTT
NC siRNA	UUCUCCGAACGUGUCACGUTT	ACGUGACACGUUCGGAGAATT

### Western blotting

The experiment was performed as per the standard protocol. Briefly, a nuclear protein and cytoplasmic protein extraction kit (Beyotime, P0027) was used to extract nuclear protein. RIPA lysis solution (Beyotime, P0013B) containing PMSF (Nanjing Wohong, 329-98-6) was used to extract the total protein. Protein concentrations were measured using the Bradford method (Bio-Rad no. 5000006). Of note, 30 μg of protein was mixed with 5 × loading buffer, and the samples were separated using a 10% sodium dodecyl sulfate–polyacrylamide gel electrophoresis (10% SDS–PAGE). The protein bands were transferred to the PVDF membrane (Bio-Rad no. 162-0177). The membrane was blocked with 4% skim milk containing 0.1% tween-20 for 1 h. The following primary antibodies was added: YAP1 antibody (1:1000, affinity, AF6328), SLC1A5 (1:2000, abcam, ab237704), GLS1 (1:2000, abcam, ab156876), histone H3 (1:2000, abcam, ab1791), GAPDH antibody (1:1000, abcam, ab9485), and the membrane was incubated at 4 °C overnight. HRP-labeled secondary antibody (Dianova, Hamburg, Germany) was added and the membrane was incubated at room temperature for 2 h. The membrane was treated with ECL developer solution (Bio-Rad no. 170-5060) and placed in the GelDoc imaging system (Bio-Rad) for visualization. The protein levels were normalized using the internal reference protein GAPDH or histone H3.

### Dual-luciferase reporter gene assay

The experiment was performed as per the standard protocol. Briefly, the cells were transfected with 8 × GTIIC-luciferase (Addgene, 34615), and then the Bright-Lumi™ firefly luciferase reporter gene detection kit (Beyotime, RG051S) was used. The cells were lysed, centrifuged at 12,000 rpm for 5 min, and the supernatant was obtained for assay. The fluorescence analyzer (Shijiazhuang Compson Technology Co., Ltd., KPS-QQ80) was used to detect firefly luciferin enzyme activity. The transcription activity of YAP/TAZ in each group was calculated using the luciferase activity of the vector group as 100% as reference.

### GLS1 activity

The enzymatic activity was calculated by measuring the amount of ammonia generated from glutamine catalyzed by GLS1 by using the indophenol blue colorimetric method. The operations followed the standard protocol. Briefly, the cells were disrupted by ultrasonic waves and centrifuged at 12,000×*g* for 15 min at 4 °C, and the supernatant was collected. The protein concentration and GLS1 activity were detected and calculated according to the manual of the GLS activity detection kit (Solarbio, BC1450).

### GPX4 activity

The operations followed the standard protocol. Briefly, the lysis buffer (100 mM Tris, pH 7.6, 5 mM EDTA, 1 mM NaN_3,_ and 0.1% Triton-X 100 without oxide) and the cells were mixed; the lysates were supplemented with 0.6 U/mL of glutathione reductase (Sigma, G3664), 0.2 mM NADPH (Sigma, N7505), 3 mM GSH (Sigma, G4251), and 200 mM cumene hydroperoxide (Sigma, 247502), and the GPX4 enzymatic activity was calculated by measuring the NADPH turnover at 340 nm.

### GSH level

The experiment was performed as per the standard protocol. Briefly, the working solution and samples or standard were mixed and incubated at room temperature for 5 min. Then, 50 μL of 0.5 mg/mL NADPH solution was added. The microplate reader was used to measure the optical density (OD) of samples and standard at 412 nm and the total glutathione was calculated. The content of GSSG was determined using GSH cleanup reagents to remove GSH from the sample. The level of GSH was calculated by subtracting the content of GSSG from the amount of total glutathione (GSSG + GSH).

### Glutamine levels

A glutamine detection kit (Nanjing Jiancheng, A073-1-1) was used to detect glutamine. The operations followed the standard protocol. Briefly, the working solution and cell culture supernatant or the standard were mixed and incubated at 37 °C for 15 min. The OD at 630 nm was detected, and the sample glutamine concentration was calculated based on the OD at 630 nm of the standard.

### Glutamate levels

A glutamate detection kit (Nanjing Jiancheng, A074-1-1) was used to detect glutamate concentration. The experiments were performed as per the standard protocol. Briefly, the cells were collected and the protein concentration was detected using the Bradford method (Bio-Rad no. 5000006). The sample or standard and the working solution were mixed and incubated at 37 °C for 40 min. The OD was detected at 340 nm. The glutamate concentration in samples was calculated based on the OD of the standard at 340 nm.

### Lipid peroxidation

The operations followed the standard protocol. Briefly, the transfected cells in the logarithmic growth phase were separated into a single cell, evenly inoculated into a 12-well cell plate, and cultured at 37 °C and 5% CO_2_. The probe C11-BODIPY (Invitrogen, D3861) was diluted with serum-free medium to a final concentration of 5 μM. Then, 1 mL of working solution was added to each well, and the mixture was incubated at 37 °C for 20 min in the dark. After washing twice with serum-free medium, the cells were detected using flow cytometry (BD Accuri C6), and the results were analyzed using the Flowjo software.

### Malondialdehyde (MDA) levels

The content of MDA was detected using the thiobarbituric acid (TBA) method as per the manufacturer’s protocol (Nanjing Jiancheng Bioengineering Institute, A003-4-1). The experiments were performed as per the kit’s manual. Briefly, the cells were collected and the protein concentration was detected using the BCA method. The working solution was added to the cell extract or standard. The mixture was warmed under a water bath at 95 °C for 40 min. The supernatant was collected and OD was measured as 530. The MDA concentration was calculated by comparing it with the results of the standard.

### ROS levels

The experiments were performed following the standard protocol. Briefly, the cells were collected, and 1 mL of diluted DCFH (Beyotime, S0033S-1) was added to the cells. The mixture was incubated at 37 °C for 21 min. Cells were washed thrice using a serum-free medium. The cell pellet was resuspended in 500 μL of PBS and stimulated by ROS positive control (Beyotime, S0033S-2) for 20 min. Lastly, the mixture was detected using a flow cytometer.

### Calcein–AM/propidium iodide (PI) staining

The staining was performed as per the manufacturer’s protocol. Briefly, the cells were collected, 100 μL of Calcein–AM/PI detection working solution (Calcein/PI Cell Viability and Cytotoxicity Detection Kit, Beyotime, C2015L) was added, and the mixture was incubated at 37 °C for 30 min in the dark. The staining effect was observed under a fluorescence microscope (Calcein-AM is green fluorescence, Ex/Em = 494/517 nm; PI is red fluorescence, Ex/Em = 535/617 nm).

### Statistics analysis

All statistical analyses were performed using GraphPad Prism9.0. The quantitative data were expressed as the mean ± standard deviation (SD). The significance between the two groups was analyzed by paired Student's *t*-test. The significance between multiple groups was determined by one-way analysis of variance (ANOVA) and LSD post-hoc multiple comparison test. *p* < 0.05 was considered to indicate statistical significance.

## Results

### YAP1 was highly expressed in CRPC tissue

**Fig. 1:**
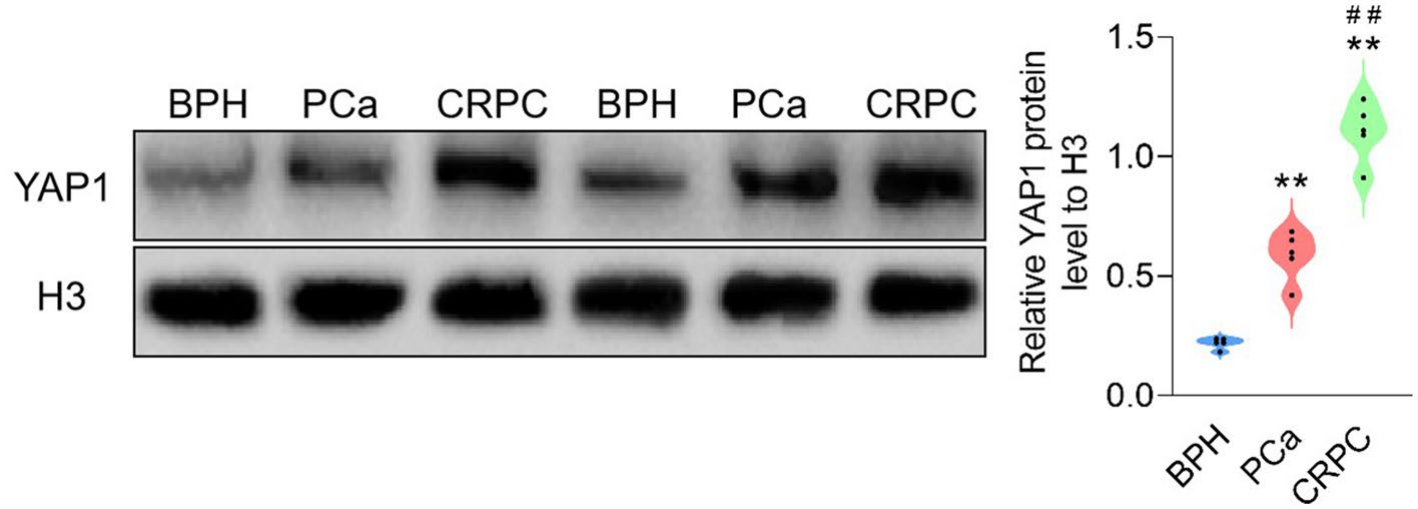
High level of YAP1 in CRPC. The levels of YAP1 in the nucleus of BPH, PCa and CRPC clinical samples were detected by Western blot and analyzed, *n* = 5. **p < 0.01 VS BPH, ##*p* < 0.01 VS PCa

We collected five tissue samples from patients with BPH and PCa who did and did not progress to CRPC and recorded their clinicopathological characteristics (Table 2). YAP1 levels in the nuclear protein of the tissue samples that did and did not progress to CRPC were significantly higher than that in the tissue samples of BPH; these levels were higher in the tissue samples that progressed to CRPC than in those that did not progress to CRPC (Fig. 1). These results suggested that YAP1 might participate in promoting the malignant progression of the disease. Thus, we focused on CRPC considering it as a refractory type of PCa with higher YAP1 levels.

**Table 2:** Information about the clinicopathological data of the patients

	BPH (*n* = 5)	PCa (*n* = 5)	CRPC (*n* = 5)
Age (year)	68.00 ± 6.24	67.80 ± 9.04	71.00 ± 11.73
Gender			
Male	5	5	5
Gleason score			
3 + 3		1	
3 + 4		2	
4 + 3		2	
4 + 5			3
5 + 4			2
Metastasis			
No		5	
Yes			5
PSA (ng/mL)	4.60 ± 2.66	9.15 ± 2.37	128.09 ± 124.23
TNM stage			
T2b		1	
T2c		4	
T4N1M1			2
T3bN1M0			1
T3bN1M1			1
TXNXM1			1

### YAP1 promotes glutamine uptake and metabolism to GSH in CRPC cells

**Fig. 2:**
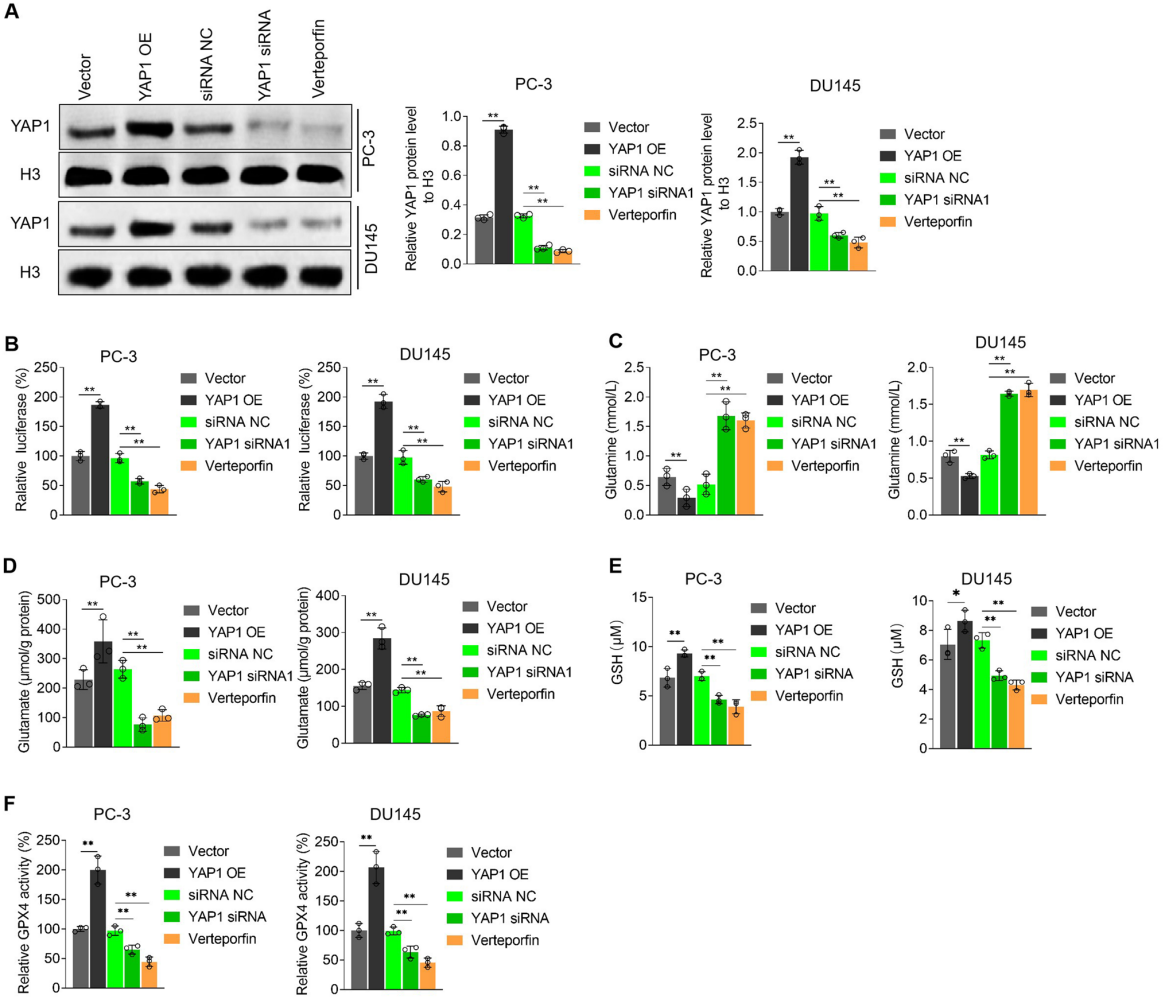
YAP1 promotes glutamine uptake and metabolism to GSH in CRPC cells. **A** Western blot detection the level of YAP1 in nucleus and quantitative statistical analysis, **B** the transcriptional activity of YAP1 in PC-3 and DU145 was confirmed by dual-luciferase reporter gene assay, **C** quantitative analysis of extracellular glutamine, **D** detection of intracellular glutamate content; used commercial kits detects GSH levels (**E**) and GPX4 activity (**F**). Verteporfin concentration 1 μM, treated for 24 h, *p < 0.05, **p < 0.01

We used the CRPC cell lines PC-3 and DU145 to determine whether YAP1 regulated glutamine uptake and metabolism in CRPC. The western blot and dual-luciferase reporter gene assays showed that YAP1 overexpression increased YAP1 levels and transcriptional activity, whereas YAP1 expression inhibition or verteporfin usage, a YAP1 and TEAD interaction inhibitor, decreased YAP1 levels and transcriptional activity (Fig. 2A and B). Furthermore, YAP1 overexpression reduced extracellular glutamine levels and increased intracellular glutamate content, whereas YAP1 activity inhibition led to the opposite results (Fig. 2C and D). Additionally, YAP1 overexpression increased GSH levels and GPX4 activity, whereas YAP1 activity inhibition significantly reversed the results (Fig. 2E and F). These results indicated that YAP1 promoted cellular glutamine uptake and metabolism to GSH.

### YAP1 inhibits ferroptosis in CRPC cells

**Fig. 3:**
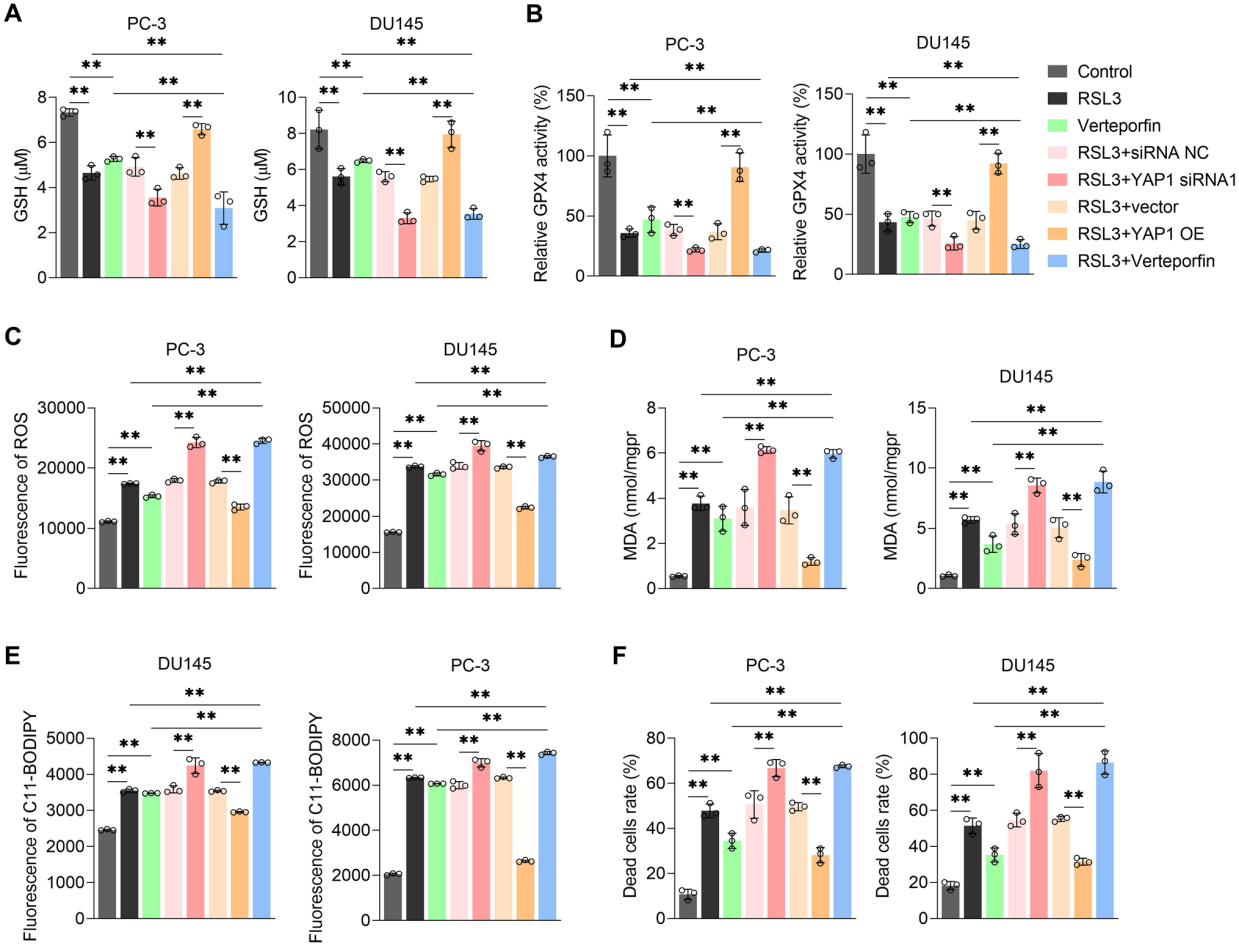
YAP1 inhibits ferroptosis in CRPC cells. Used commercial kits detects GSH levels (**A**) and GPX4 activity (**B**), **C** ROS level was detected by DCFH fluorescent probe, **D** used commercial kit detects MDA level, **E** Lipid oxidation level displayed by BODIPY™ 581/591 C11 lipid oxidation probe, **F** Calcein-AM/PI analysis of the proportion of dead cells in each group. RSL3 concentration 5 μM, treated for 24 h, **p < 0.01

GPX4 is the core of the antioxidant system and inhibits ferroptosis. Moreover, ferroptosis activation effectively enhances the sensitivity of CRPC to chemotherapeutic drugs. Therefore, we speculated that YAP1 affects ferroptosis in CRPC cells. To test this speculation, we used RSL3 to induce ferroptosis. Initially, we found that RSL3 reduced GSH levels and GPX4 activity in PC-3 and DU145 cells. YAP1 overexpression partially restored GSH levels and GPX4 activity, whereas YAP1 inhibition further decreased GSH levels and GPX4 activity (Fig. 3A and B). Then we found that YAP1 overexpression reduced ROS and MDA levels and BODIPY™ 581/591 C11 fluorescence intensity in PC-3 and DU145 cells, whereas YAP1 inhibition increased ROS and MDA levels and BODIPY™ 581/591 C11 fluorescence intensity (Fig. 3C–E). The calcein–AM/PI staining assay revealed that YAP1 overexpression reduced cell death extent, whereas its inhibition showed the opposite trend (Fig. 3F). These results indicated that YAP1 inhibited ferroptosis in the CRPC cells.

### Inhibition of SLC1A5 or GLS1 promotes ferroptosis in CRPC cells

**Fig. 4:**
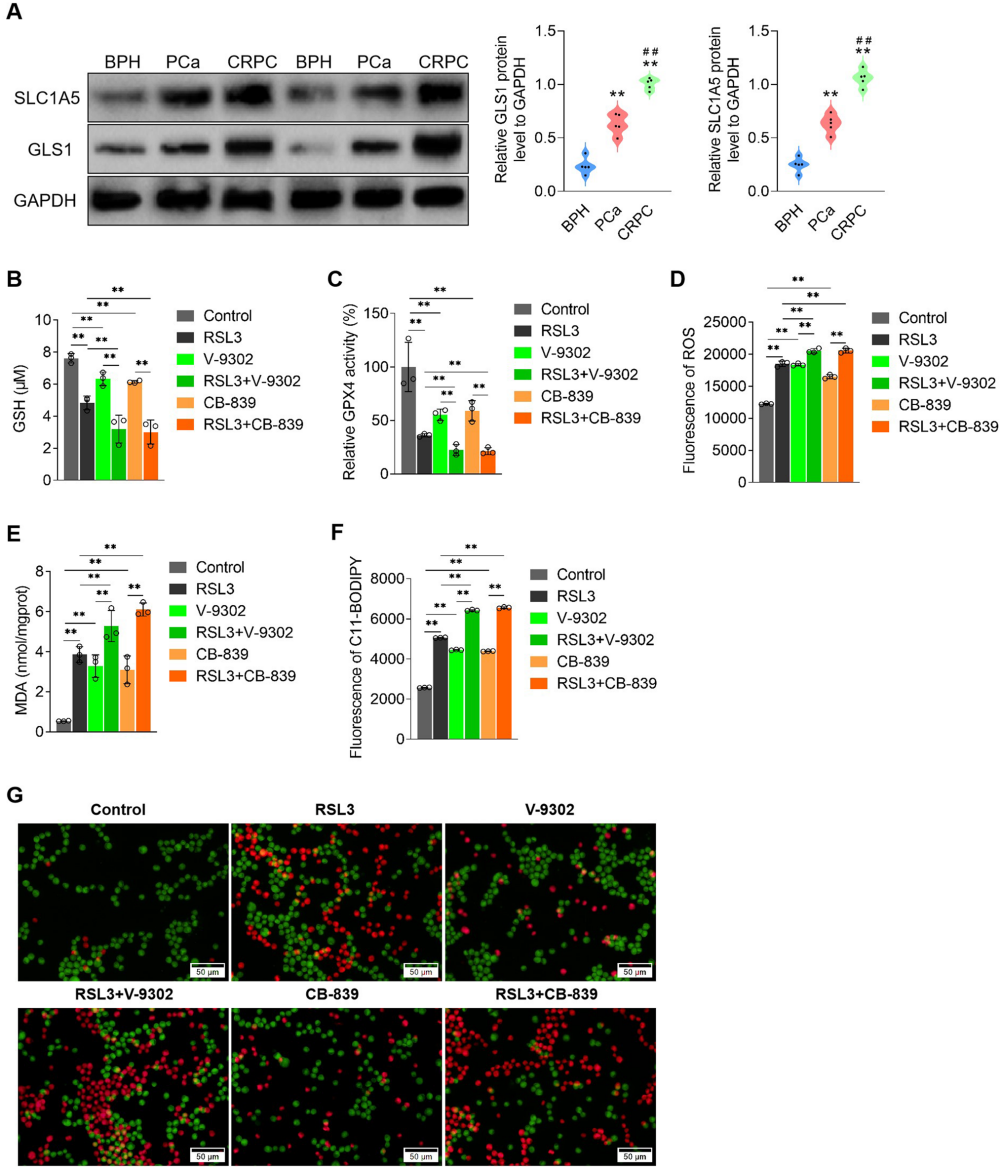
Inhibition of SLC1A5 or GLS1 promotes ferroptosis in CRPC cells. **A** The levels of YAP1 in the nucleus of BPH, PCa and CRPC clinical samples were detected by Western blot and analyzed, *n* = 5; used commercial kits detected GSH levels (**B**), GPX4 activity (**C**) and MDA levels (**E**); **D** ROS level was detected by DCFH fluorescent probe, **F** lipid oxidation level displayed by BODIPY™ 581/591 C11 lipid oxidation probe, **G** Calcein-AM/PI analysis of the proportion of dead cells in each group. V-9302 concentration 20 μM, time 48 h; CB-839 concentration 1 μM, treated for 48 h, *p < 0.05, **p < 0.01

Glutamine promotes CRPC progression, and glutamine can be metabolized to GSH to increase GPX4 activity [21, 22]. SLC1A5 and GLS1, key molecules affecting glutamine uptake and metabolism to generate GSH [23, 24], may regulate ferroptosis in CRPC cells. Thus, we first examined SCL1A5 and GLS1 levels in the tissue samples that did and did not progress to CRPC and found that the levels were significantly higher than that in BPH, whereas they were higher in the tissues that progressed to CRPC than in those that did not progress to CRPC (Fig. 4A). Then, we treated PC-3 cells with V-9302, which inhibits SLC1A5, a plasma membrane transporter that imports glutamine, and CB-839, which inhibits GLS1, an enzyme that converts glutamine to glutamate [25]. V-9302 and CB-839 decreased GSH levels and GPX4 activity (Fig. 4B and C) and increased ROS and MDA levels, BODIPY™ 581/591 C11 fluorescence intensity (Fig. 4D–F), and dead cell proportion (Fig. 4G). These results indicated that SLC1A5 or GLS1 inhibition promoted ferroptosis in the CRPC cells.

### YAP1 expression increased SLC1A5 and GLS1 levels in CRPC cells

**Fig. 5:**
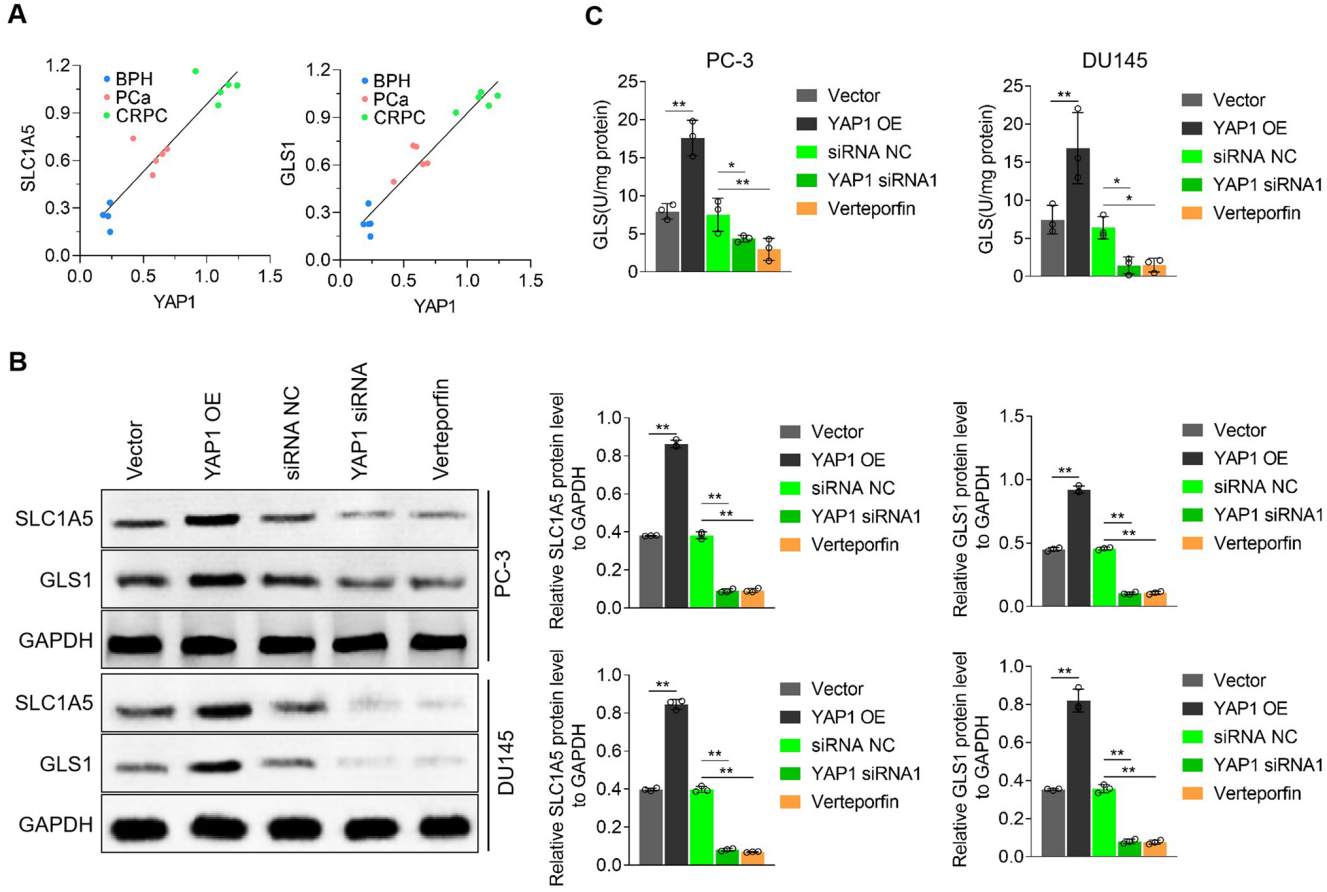
YAP1 promotes SLC1A5 and GLS1 expression in CRPC cells. **A** Correlation analysis between YAP1 level and SLC1A5 or GLS1 levels. **B** Western blot detection the level of SLC1A5 and GLS1, and quantitative statistical analysis, **C** used commercial kits detects GLS1 activity. *p < 0.05, **p < 0.01

To clarify the effect of YAP1 expression on SLC1A5 and GLS1 levels in CRPC, we first performed a correlation analysis on YAP1, SLC1A5, and GLS1 levels in the clinical samples and found that YAP1 expression was positively correlated with SLC1A5 and GLS1 levels (Fig. 5A). Further, we found that in the PC-3 and DU145 cells, YAP1 overexpression increased SLC1A5 and GLS1 levels and activities, whereas YAP1 inhibition decreased SLC1A5 and GLS1 levels and activities (Fig. 5B and C). These results indicated that YAP1 expression increased SLC1A5 and GLS1 levels in CRPC cells.

### YAP1 inhibits ferroptosis in CRPC cells by promoting glutamine uptake and metabolism

**Fig. 6:**
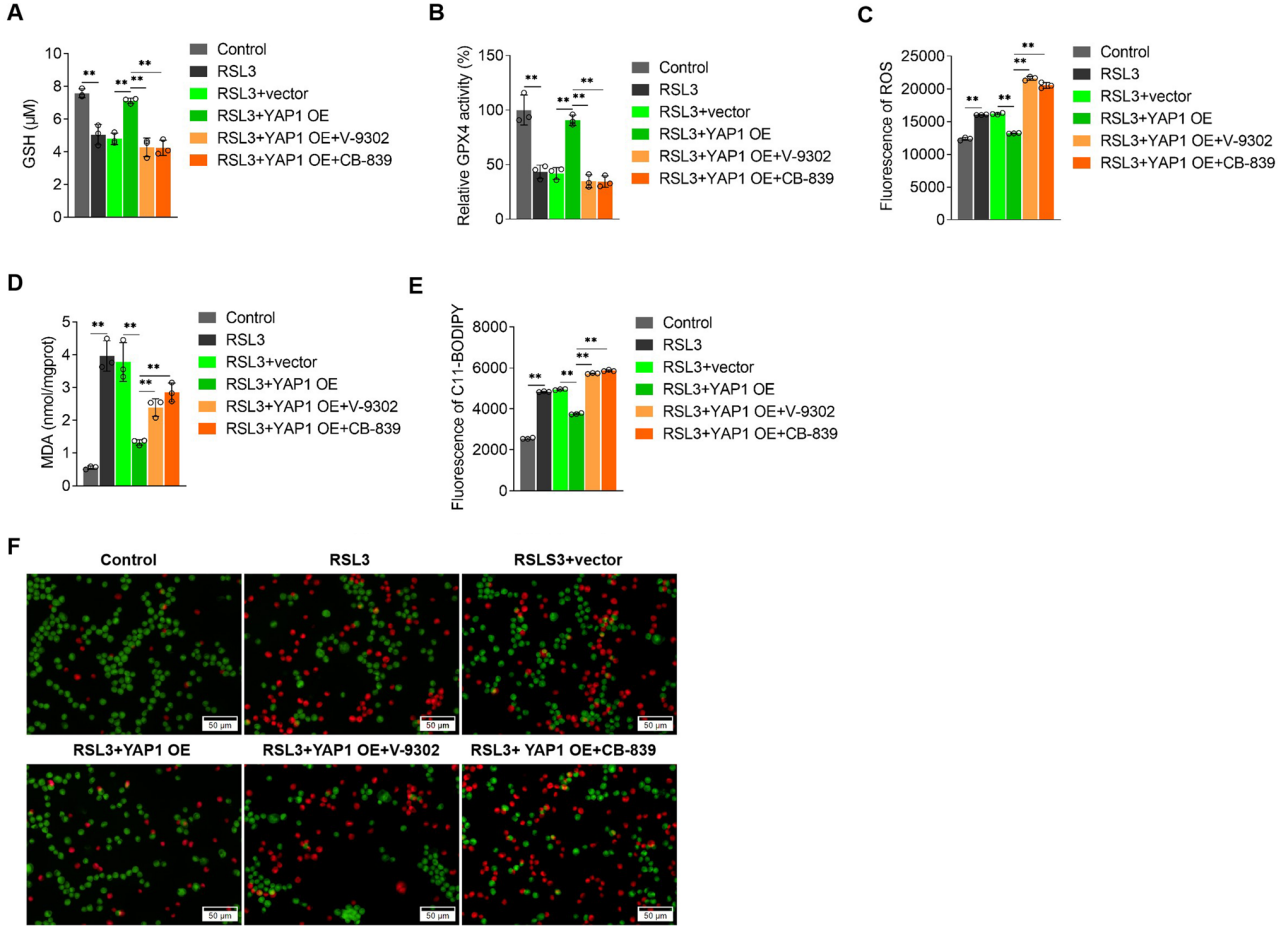
YAP1 inhibits ferroptosis in CRPC cells by promoting the expression of SLC1A5 and GLS1. Used commercial kits detected GSH levels (**A**), GPX4 activity (**B**) and MDA levels (**D**), **C** ROS level was detected by DCFH fluorescent probe, **E** lipid oxidation level displayed by BODIPY™ 581/591 C11 lipid oxidation probe, **F** Calcein-AM/PI analysis of the proportion of dead cells in each group. **p < 0.01

Because YAP1 expression can increase SLC1A5 and GLS1 levels in CRPC cells and YAP1, SLC1A5, and GLS1 inhibit ferroptosis in CRPC cells, we investigated whether YAP1 inhibits ferroptosis in CRPC cells by increasing SLC1A5 and GLS1 levels. We found that SLC1A5 or GLS1 inhibition decreased the positive regulatory effect of YAP1 on GSH levels and GPX4 activity (Fig. 6A and B) and increased ROS and MDA levels, BODIPY™ 581/591 C11 fluorescence intensity (Fig. 6C–E), and dead cell proportion (Fig. 6F). There results indicated that YAP1 inhibited ferroptosis in the CRPC cells by increasing SLC1A5 and GLS1 levels.

## Discussion

An effective treatment option is not available for CRPC, a type of PCa. Ferroptosis activation enhances the sensitivity of CRPC cells to 5-FU and docetaxel. YAP1 is highly expressed in CRPC cells; however, its role in regulating ferroptosis in CRPC cells and the underlying specific mechanism remain unclear. Herein, we found that high YAP1 expression in CRPC was positively correlated with SLC1A5 and GLS1 levels. At the cellular level, YAP1 expression increased the levels of SLC1A5, GLS1, and GSH, the activity of GPX4, the cellular uptake of glutamine, metabolized glutamine to glutamate, inhibited the effects of RSL3 on increasing the levels of ROS, MDA, and lipid peroxidation, and decreased the proportion of cell death, thereby antagonizing cell ferroptosis.

Ferroptosis is a type of programmed cell death characterized by ROS accumulation and lipid peroxidation. On the molecular level, cysteine availability, GSH biosynthesis, and proper GPX4 functioning are the core of ferroptosis, whereas conditions that promote GPX4 inhibition/destabilization sensitize or even trigger ferroptotic cell death [26]. Studies have shown that drug-resistant cancer cells, including breast, lung, and ovarian cancer and melanoma, exhibit mesenchymal-like gene expression and can be sensitized to cell death using GPX4 inhibitors; however, this phenomenon does not occur in normal cells and tumor cells that are not treated with GPX4 inhibitors [27, 28]. CRPC cells are not sensitive to androgen-deprivation or targeted androgen drugs and exhibit the phenomenon of increased stemness. RSL3 can be used to inhibit GPX4 activity, thereby inhibiting cell viability, clone formation, migration ability, and in vivo tumor growth and promoting cell death [27, 29]. We found that RSL3 increased the rate of CRPC cell death, similar to the finding of Tanya Stoyanova et al. and Stuart L Schreiber et al. The targeted inhibition of GPX4 is an ideal way to intervene in CRPC progression. An understanding of the mechanism of the regulatory activity of GPX4 in CRPC cells may help find more effective targets for inhibiting CRPC.

In PCa, AR normally recruits the histone methyltransferase EZH2 and the DNA methyltransferase DNMT3a to the YAP1 promoter, which increases the level of promoter methylation and inhibits YAP1 expression. PCa is treated by ADT, for example, the AR antagonist bicalutamide or enzalutamide, which helps PCa to progress into CRPC, or in the case of AR mutation or suppressed AR expression, the effect of AR to inhibit YAP1 expression is weakened [30]. Additionally, a study has shown that docetaxel-based chemotherapy upregulates YAP1 expression and increases nuclear YAP1 levels in CRPC, and the recurrence rate is higher when the nuclear YAP1 level is higher [31]. The abovementioned changes are associated with the higher level of YAP1 in CRPC, suggesting that YAP1 exhibits a unique function in CRPC. Studies have shown that high levels of YAP1 are involved in promoting CRPC growth, metastasis, and drug resistance [12, 15–17]. A recent study has shown that YAP1 does not always induce ferroptosis and sometimes inhibits ferroptosis, and its role in ferroptosis is cell-context specific [18]. Most prominently, YAP activates ferroptosis in mesothelioma, and the underlying mechanism is that unphosphorylated YAP/TAZ translocates to the nucleus to interact with transcription factors, such as TEAD1–4, to transcribe target genes [32, 33]. Target genes of YAP that regulate ferroptosis in various ways include GPX4, acyl-coenzyme A (CoA) synthetase long-chain family member (ACSL4), transferrin receptor (TFRC), NADPH oxidase 2 (NOX2), and NOX4 [34]. GPX4 is a GPX family member that transforms lipid peroxides to lipid alcohols using GSH as a cofactor, thereby suppressing ferroptosis [35]. Additionally, RSL3 inhibits GPX4, and the loss or inhibition of GPX4 induces ferroptosis in cancer cells [29]. In non-small cell lung cancer, hypoxia-inducible factor (HIF)-1α inhibits ferroptosis by activating the Hippo–YAP signaling pathway, and YAP1 upregulates GPX4 expression [36]. However, the role of YAP1 in CRPC ferroptosis has not been investigated. Herein, we found that YAP1 increased GSH levels, thereby increasing GPX4 activity and inhibiting ferroptosis in CRPC. Furthermore, the inhibition of YAP1 expression or transcriptional activity increased the sensitivity of CRPC cells to ferroptosis. We are the first to establish a relationship between YAP1 and ferroptosis in CRPC and clarify that YAP1 can inhibit ferroptosis by improving the antioxidant capacity of CRPC cells, which is different from the relationship between YAP1 and ferroptosis in colorectal cancer from the perspective of oxidation [33, 37, 38]. We further explored the role of YAP1 in CRPC and showed that YAP1 may be a more suitable target than GPX4 to increase ferroptosis, inhibit tumor recurrence and metastasis, and improve drug sensitivity.

Glutamine, the most abundant amino acid in human blood, is closely related to tumor progression. Previous studies have shown that tumor cells mainly use glucose for glycolysis and the biosynthesis of macromolecular precursors even in the presence of sufficient nutrients, which is called the Warburg effect [39]. Conversely, the latest research shows that the main glucose-utilizing cells in tumor tissues are myeloid cells and that tumor cells mainly utilize glutamine and lipids [40]. After glutamine enters tumor cells, it affects autophagy, generates α-ketoglutarate, and metabolizes glutamate to further convert into GSH [8]. A study has shown that SLC1A5 inhibition in PC-3 cells reduces glutamine uptake, oxygen consumption, and fatty acid synthesis and suppresses tumor growth and metastasis; however, α-ketoglutarate addition reduces these effects of SLC1A5 inhibition [41]. SLC1A5 is an essential transporter for glutamine uptake, and SLC1A5-mediated glutamine transport plays a key role in tumor cell metabolism, proliferation, and ferroptosis [42]. Additionally, GLS1 is the rate-limiting enzyme in glutamine catabolism [24]. Herein, we found that SLC1A5 and GLS1 inhibition decreased GSH levels and GPX4 activity and improved CRPC cell sensitivity to RSL3-induced ferroptosis, whereas YAP1 increased GSH levels and GPX4 activity in cells by increasing SLC1A5 and GLS1 levels, antagonized ferroptosis in CRPC, and promoted the role of glutamine metabolism in CRPC. We also found the mechanism by which YAP1 regulates ferroptosis. Additionally, autophagy plays an important role in CRPC [43, 44], and α-ketoglutarate generated by glutamine metabolism functions via the TCA cycle and synthesizes biological macromolecules and acts as a cofactor for the histone demethylases JmjC-KDMs and members of the DNA demethylase TET family, participating in promoting histone and DNA demethylation processes and regulating gene expression [45]. However, whether glutamine metabolism regulates CRPC progression via autophagy and epigenetic mechanisms and whether YAP1 plays an upstream role remain unknown, and we will conduct relevant studies in the future.

In conclusion, this study shows that YAP1 inhibits ferroptosis in CRPC cells by promoting glutamine uptake and conversion to glutamate, thereby increasing GSH levels and GPX4 activity. The study also suggests the underlying pathological mechanism. YAP1 inhibits ferroptosis in CRPC; thus, ferroptosis can be activated by inhibiting YAP1 for treating CRPC. Existing studies have shown that zoledronic acid inhibits the entry of YAP into the nucleus by maintaining its phosphorylated state [32]. Based on the findings that verteporfin inhibits binding between YAP and TEAD to limit its transcriptional activity, JQ-1 inhibits YAP-mediated transcription by inhibiting bromodomain-containing protein 4, an essential component of the YAP–TEAD complex [46]. Thus, the present study provides insights and a theoretical basis for developing appropriate strategies to manage CRPC.

## Data Availability

The authors confirm that the data supporting the findings of this study are available within the article [and/or its supplementary materials].
